# Using Postbiotics from Functional Foods for Managing Colorectal Cancer: Mechanisms, Sources, Therapeutic Potential, and Clinical Perspectives

**DOI:** 10.3390/microorganisms13061335

**Published:** 2025-06-09

**Authors:** Teresa D’Amore, Cinzia Zolfanelli, Vincenzo Lauciello, Alessio Di Ciancia, Alessio Vagliasindi, Slim Smaoui, Theodoros Varzakas

**Affiliations:** 1 Laboratory of Preclinical and Translational Research, IRCCS CROB, Centro di Riferimento Oncologico della Basilicata, 85028 Rionero in Vulture, Italy; teresa.damore@crob.it (T.D.); cinzia.zolfanelli@crob.it (C.Z.); vincenzo.lauciello@crob.it (V.L.); alessio.diciancia@crob.it (A.D.C.); 2 Unit of Abdominal Oncological Surgery, IRCCS CROB, Centro di Riferimento Oncologico della Basilicata, 85028 Rionero in Vulture, Italy; alessio.vagliasindi@crob.it; 3 Laboratory of Microbial and Enzymes Biotechnology and Biomolecules (LMEBB), Centre of Biotechnology of Sfax (CBS), University of Sfax-Tunisia, Road of Sidi Mansour Km 6, P.O. Box 1177, Sfax 3018, Tunisia; slim.smaoui@cbs.rnrt.tn; 4 Department of Food Science and Technology, University of the Peloponnese, 24100 Antikalamos, Kalamata, Greece

**Keywords:** postbiotics, colorectal cancer, carcinogenesis, functional food, advanced preclinical models, disease modelling, chemoprevention

## Abstract

Postbiotics, defined as a preparation of inanimate microorganisms and/or their components, including metabolic byproducts, have gained recognition as promising modulators of gut health and disease, offering advantages over probiotics in terms of safety, stability, and formulation. This systematic review investigates the therapeutic potential of postbiotics derived from functional foods in the context of colorectal cancer (CRC), a leading cause of cancer-related mortality worldwide. Despite encouraging preclinical findings, translation into clinical practice remains limited due to a paucity of robust human trials, revealing a significant gap and the need for further translational research. Key bioactive categories of postbiotics are described, alongside their anti-inflammatory, immunomodulatory, and chemopreventive mechanisms. Through comprehensive literature mapping, this review uniquely categorizes research according to the experimental models employed, i.e., in vitro, in silico, in vivo, and ex vivo, and advanced models such as organoids and organ-on-chip platforms. The latter offers greater physiological relevance by closely mimicking human tissue architecture and microenvironment. These models help demonstrate how postbiotics may influence tumorigenesis through mechanisms involving inflammation, apoptosis, epigenetic regulation, and the maintenance of gut barrier integrity. Finally, the review summarizes recent innovations in their delivery strategies and calls for comprehensive mechanistic studies and high-quality clinical trials to validate postbiotics as safe and effective adjuncts in CRC prevention, therapy, and management.

## 1. Introduction

In the last two decades, studies and research on microbiota and its role and therapeutic potential in human health and disease have increased. The microbiota has been referred to as the “hidden organ”, with more than 100 trillion microorganisms inhabiting various body districts and contributing a greater percentage of genetic material than the entire human genome. Among the different microbiotas (e.g., respiratory, skin, oral, gut, vagina, etc.), the gut microbiota is considered particularly critical due to its profound influence on host metabolism, immunity, and disease susceptibility [1]. Increasing evidence supports the association between the gut microbiota dysbiosis, defined as an imbalance in the composition and species diversity of resident commensal microorganisms, associated with changes in metabolic activities or functions, and the pathogenesis of several diseases (i.e., cardiovascular and respiratory diseases, many types of cancer, diabetes and metabolic disorders, polycystic ovary syndrome, inflammatory bowel diseases, neurological disorders, chronic kidney diseases, and liver diseases) [2,3,4,5,6,7,8,9].

Food intake, nutritional status, dietary habits, and supplementation are key modulators of gut microbiota composition and activity. These factors not only determine microbial diversity and metabolic output but also critically impact host physiological functions, immune homeostasis, and disease susceptibility [10,11,12].

**Figure 1:**
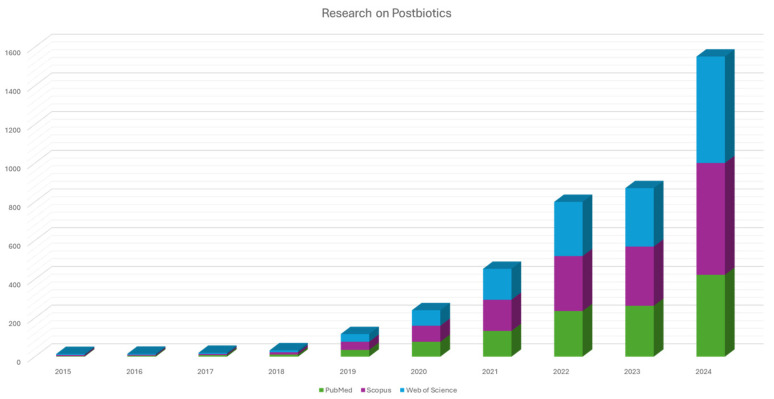
Trends of scientific publications on postbiotics over the last decade (2015–2024), based on data extracted from PubMed, Scopus, and Web of Science. The figure reflects the increasing number of studies focused on postbiotics, highlighting the growing interest and recognition of their therapeutic potential, especially in areas such as gut health, inflammation, and cancer.

Recognized modulators of gut health are prebiotics (non-digestible food components, such as dietary fibers and oligosaccharides that promote the growth of beneficial commensal microorganisms) and probiotics, i.e., live microorganisms that, when administered in adequate amounts, confer a health benefit and can directly enrich and stabilize the gut microbial community. While probiotics and prebiotics have traditionally dominated this field, emerging attention is now directed toward postbiotics [13,14]. The International Scientific Association for Probiotics and Prebiotics (ISAPP) recently defined postbiotics as “a preparation of inanimate microorganisms and/or their components that confers a health benefit on the host” [15,16,17]. Studies on postbiotics have increased exponentially in the last few years, as evidenced in Figure 1, which shows the number of studies on postbiotics extracted from PubMed, Scopus, and Web of Science in the last decade (2015–2024). This upward trend correlates with the recent standardization of terminology, formalized only in 2019 by ISAPP, and criteria by scientific bodies, as well as improved mechanistic insights and advances in biotechnology enabling broader application of non-viable microbial components. Postbiotics have several advantages over probiotics, including greater safety, enhanced stability, and easier formulation. Their inanimate nature eliminates concerns regarding bacterial translocation, infection risk, and the transfer of antibiotic resistance genes, issues particularly critical for immunocompromised or critically ill individuals. Moreover, postbiotics can exert diverse biological activities, such as anti-inflammatory, immunomodulatory, antioxidant, and anti-proliferative effects, which are increasingly being investigated in the context of chronic diseases [18,19,20].

In this complex scenario, this study aims to collect and review the role of functional foods that serve as rich sources of postbiotics, either through natural fermentation processes or via microbial biotransformation of dietary components, in a multifactorial disease like colorectal cancer (CRC) [21,22]. Despite recent advancements in the comprehension of its cellular and molecular mechanisms, CRC remains a major global health concern, ranking as the second leading cause of cancer-related deaths and the third most frequently diagnosed malignancy after lung and breast cancer. According to the Global Cancer Observatory (GCO) and the International Agency for Research on Cancer (IARC), nearly two million new cases of CRC were reported, with over 900,000 deaths. They account for approximately 9.3% of total cancer-related deaths. Mortality rates are notably higher in men than in women [23].

CRC is a complex disease associated with genetic predisposition and environmental risk factors. Key contributors include family history, hereditary CRC syndromes, inflammatory bowel diseases, dietary habits, fiber deficiency, and obesity. Additionally, smoking, sedentary lifestyles, and aging have been strongly correlated with CRC incidence. The disease is often asymptomatic in its early stages, leading to late-stage diagnoses, which significantly impact treatment efficacy and patient survival [24]. Early detection through screening programs has proven to be a crucial strategy for reducing CRC incidence and mortality. Data from the European Commission (EC) CRC Statistics show that countries with long-standing, population-wide screening programs experienced a significant decline in CRC incidence between 2000 and 2017, whereas those without widespread screening initiatives reported either stable or rising cases. Among various screening tools, colonoscopy remains the most effective method for detecting and removing precancerous adenomas. However, due to its invasive nature, associated discomfort, and potential complications, its use as a routine screening method remains limited [25]. Pharmacological therapeutic interventions, both in a presurgical phase (neoadjuvant therapy) and after mass resection (adjuvant therapy), may have some limitations, such as severe adverse effects and chemoresistance, necessitating the exploration of novel complementary strategies [26].

For these reasons, this review aims to summarize the sources and classes of postbiotics from functional foods and then describe, using the methodologies of systematic review and meta-analysis, the studies on postbiotics in CRC. Mechanisms of action, including anti-inflammatory actions, modulation of tumor cell signaling pathways, and reinforcement of intestinal barrier integrity, through which postbiotics derived from functional foods may offer a novel, safe, and effective adjunct in CRC management, are presented. This review discusses properties, technological functions, and health implications of postbiotics in CRC in detail and lays special emphasis on research carried out using advanced preclinical study models (e.g., organoids, spheroids, organs-on-chip, ex vivo models, etc.), as well as clinical microbiota-targeted interventions. The translation of postbiotic-based interventions into clinical practice requires careful consideration of safety profiles, standardized production methods, and regulatory approval processes, since no specific regulations currently cover postbiotics [13,18]. While postbiotics demonstrate favorable stability and risk profiles compared to live biotherapeutics, challenges persist in the characterization of active components, all of which require individual safety and efficacy assessments, and in establishing robust clinical evidence of efficacy. As the field progresses, rigorous scientific validation and the establishment of clear regulatory frameworks will be pivotal for their successful integration into cancer prevention and treatment strategies.

## 2. Sources and Classification of Postbiotics

**Figure 2:**
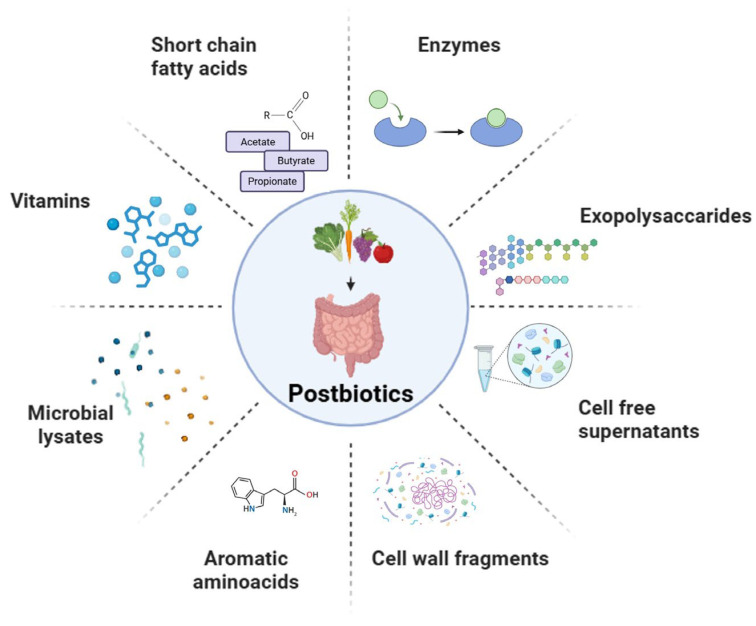
Postbiotic bioactive metabolites.

Although a harmonized classification has yet to be established, postbiotics are generally categorized into several types, including short-chain fatty acids, exopolysaccharides, cell wall fragments, cell-free supernatants, enzymes, and a variety of other metabolic byproducts (Figure 2) [27,28,29,30]. They play a catalytic role in the preservation of the stability of microbial communities and the facilitation of host–microbe interactions [8,31]. Based on their chemical structure and function, postbiotics can also be categorized into carbohydrates (e.g., teichoic acids (TAs) and galactose-rich polysaccharides), proteins, lipids (including butyrate, acetate, and dimethyl acetyl-derived plasmalogens, which are membrane phospholipids), vitamins (primarily B-group vitamins), organic acids (such as 3-phenyllactic acid and propionic acid), and other complex biomolecules (including lipoteichoic acids (LTAs) and peptidoglycan-derived muropeptides) [32,33,34].

### 2.1. Short-Chain Fatty Acids

Short-chain fatty acids (SCFAs), saturated aliphatic organic acids containing between one and six carbon atoms, represent a key group of microbial metabolites. They are introduced through diet and primarily produced by intestinal bacteria via the fermentation of non-digestible carbohydrates such as dietary fibers [35,36]. The most physiologically significant comprise acetate, propionate, and butyrate, which account for approximately 85–95% of the total SCFAs present in the colon [37,38,39,40]. Acetate is the most abundant SCFA, generated as an end-product of fermentation by enteric bacteria. It can also be produced from formate through the Wood–Ljungdahl pathway by hydrogenotrophic bacteria such as *Acetobacterium woodii* [41]. Through the fermentation of nondigestible carbohydrates, *Lactobacillus* and *Bifidobacterium* can generate organic acids like lactic acid. Under normal gut conditions, there is cross-feeding between bacteria, whereby species such as *Eubacterium hallii* further convert lactic acid into SCFAs [42].

KetoA (10-oxo-12(Z)-octadecenoic acid), a metabolite of linoleic acid synthesized by lactic acid bacteria (LAB), constitutes another interesting postbiotic. This metabolite was shown to be efficient at enhancing energy expenditure and metabolic health with therapeutic potential for the management of metabolic disorders [43].

Prebiotics (e.g., inulin and fructooligosaccharides) are fermented by the gut microbiota to produce SCFAs [44]. Under heterofermentative conditions, probiotics also use the phosphoketolase pathway as an alternative pathway for SCFA biosynthesis [45]. For example, *Bifidobacteria* have varied metabolic flexibility based on the availability of nutrients. Under carbohydrate excess, they produce acetate and lactate; on the contrary, under carbohydrate limitation, they shift metabolic strategy, producing acetate and formate via committed fermentation pathways.

### 2.2. Exopolysaccharides

On the basis of their chemical structure, exopolysaccharides (EPSs) are generally divided into two broad categories: (i) homopolysaccharides, including levan, cellulose, pullulan, curdlan, and dextran, which are composed of one type of monosaccharide unit; and (ii) heteropolysaccharides, such as gellan, xanthan, kefiran, and galactan, which have repeating units of two or more different monosaccharides [46].

The EPS’ structural complexity was affected by key microbial processes like biofilm formation, adhesion, protection against environmental stresses, and retention of nutrients. EPS also arbitrate key interactions with host organisms, including immunomodulation, colonization, and the formation of symbiotic or pathogenic relationships [47]. Their structural diversity enables EPSs to portray a range of health-beneficial activities, including antimutagenic, antitumor, and immunomodulatory activities. Many LAB-derived EPSs are strong antioxidant, antibacterial, antihypertensive, anti-inflammatory, and antiviral agents, and thus are excellent candidates for application in nutraceuticals, functional foods and therapy regimens [48]. Certain EPSs produced by *Lactobacillus* strains, selected from fermented durian fruit, have shown antioxidant and antimicrobial activities, corroborating their health-promoting effects. In addition, the EPS kefiran was shown to beneficially affect lipid metabolism and manage atherosclerosis [49]. EPSs showed very good antioxidant activity, supported by their ability to improve glutathione peroxidase, superoxide dismutase, and catalase activities of these key antioxidant enzymes. Additionally, EPS treatment was able to reduce the levels of lipid peroxidation in both the serum and liver tissues of mice, which indicates their potential in protecting against and maintaining metabolic health and oxidative damage [50,51].

### 2.3. Enzymes

Enzymes are classified on the basis of their catalytic action into six broad categories: transferases, oxidoreductases, lyases, hydrolases, isomerases, and ligases, each catalyzing a different type of biochemical reaction [52]. Their GRAS status (Generally Recognized As Safe) makes them highly suitable for application in food, pharmaceutical, and biotechnological processes [53]. Kim et al. reported that two strains of *Lactobacillus fermentum* produced high glutathione peroxidase concentrations with effective in vitro antioxidant activity. *Lb. acidophilus* KCTC 3111 showed the strongest inhibition of lipid peroxidation (50% in whole cells, 65% in lysates) and strong hydroxyl radical scavenging activity. *L. brevis* KCTC 3498 showed the highest glutathione peroxidase activity [54]. Similarly, proteases from *Bacillus* species are gaining increased attention as postbiotic entities due to their potent bioactivities and stability under gastrointestinal conditions. These enzymes, which are excreted via fermentation or microbial metabolism, continue to be active in the absence of viable cells. *Bacillus subtilis* and *Bacillus licheniformis* are well recognized to produce healthy neutral and alkaline proteases that are extremely heat-, pH-, and digestive enzyme-resistant, thereby qualifying them as potential candidates for oral delivery in functional foods and supplements. These proteases exert health benefits through the generation of bioactive peptides with antioxidant, antimicrobial, anti-hypertensive, and immunomodulatory activities. Moreover, they can enhance protein digestibility, modulate gut microbiota, and support intestinal barrier function—the main ways in which postbiotics exert beneficial effects. The use of *Bacillus* proteases as postbiotics is a growing field of research within microbiome-based therapies and functional nutrition, particularly since they are simple to produce, have GRAS status, and their industrial scalability has already been demonstrated [30]. Catalase is an enzyme crucial for lowering oxidative stress, a factor in numerous health disorders. It was demonstrated that strains that produce catalase, i.e., *Lactococcus lactis*, can decrease oxidative damage in the colon, thereby restricting inflammation and, in turn, the development of CRC in mice [55]. Supplemental dietary administration of exogenous catalases derived from *Penicillium notatum* in weaned pigs was shown to enhance intestinal antioxidant defenses in the pig by alleviating lipopolysaccharide (LPS)-induced oxidative damage, where LPS is a pro-inflammatory endotoxin. The supplement not only reduced oxidative stress markers but also beneficially modulated the gut microbiota population [56]. Similarly, the dietary addition of catalase in broiler chickens challenged with deoxynivalenol (DON), a common mycotoxin, led to the reduction of intestinal oxidative stress, improvement in villus morphology, and reestablished microbiota homeostasis. Such effects suggest that catalase performs effectively as a postbiotic by blocking ROS-mediated damage [57,58].

### 2.4. Cell Wall Fragments

Recent studies have highlighted the noticeable role played by probiotic bacterial cell wall fragments in gut health promotion and immune modulation [59]. Peptidoglycan fragments, such as muramyl dipeptide, were demonstrated to modulate immune responses by regulating cytokine secretion and activating Toll-like receptors (TLRs) as pattern recognition receptors. For example, *Lactobacillus rhamnosus* CRL1505 peptidoglycan could have enhanced the resistance to *Streptococcus pneumoniae* infection by normalizing the serum level of pro-inflammatory cytokines such as TNF-α, IL-1β, and IL-6. Other than immune modulation, cell wall fragments have anti-inflammatory and antioxidant effects. As an example, lipoteichoic acid from *Lactobacillus plantarum* has been reported to induce the production of TNF-α and the phosphorylation of NF-κB-p65, p-38, and JNK, thereby regulating immune responses [59]. Their use in the formulation of functional foods or supplements may present a new opportunity for enhancing health status, especially in populations at risk of gastrointestinal diseases or chronic illnesses. Jung et al. purified LTAs from four *Lactiplantibacillus plantarum* strains—K8, K88, K5-5, and K55-5—and compared their immunostimulatory activities. The authors established that structural variations of LTAs between the strains greatly influenced their ability to modulate immune responses. More specifically, differences in glycosylation patterns, D-alanine substitutions, and molecular weights of the LTAs were found to modulate immune cell activation of macrophages [60].

### 2.5. Cell-Free Supernatants

Cell-free supernatants (CFSs) characterize a heterogeneous postbiotics class with multilayered roles in promoting health. Current investigations have stressed the efficacy of LAB-derived CFSs against a range of pathogenic microorganisms. For example, CFSs from *Lb. aacidophilus*, *Lb. rhamnosus*, and *Lactiplantibacillus plantarum* have established potent antibacterial effects against foodborne pathogens such as *Salmonella* and *E. coli*. These CFSs displayed antimicrobial activity even after lyophilization or neutralization, signifying their robustness and potential as natural preservatives in animal feed and food products [61]. Furthermore, CFSs have shown potential in combating biofilm formation, a common challenge in long-lasting contaminations and food spoilage. LAB-derived CFSs can disturb biofilm integrity and constrain the expression of virulence factors in pathogens like *P. aeruginosa*, *S. aureus*, and *Candida* species, indicating their potential in therapeutic and food safety applications. Beyond antimicrobial activities, LAB-derived CFSs possess antioxidant properties, which can be beneficial in preventing oxidative stress-related diseases. Additionally, studies have demonstrated that CFS from probiotic strains such as *Lactobacillus casei* and *Lactobacillus rhamnosus GG* can inhibit CRC cell invasion by modulating matrix metalloproteinase-9 (MMP-9) activity and enhancing tight junction protein levels, thereby contributing to cancer metastasis prevention [62].

### 2.6. Functional Foods as Sources of Postbiotics

#### 2.6.1. Sauerkraut (Fermented Cabbage)

Fermented cabbage, commonly known as sauerkraut (also called suan cai, curtido, and other regional names), produces a consistent metabolomic profile rich in bioactive compounds recognized as important postbiotics. These include metabolites such as D-phenyl-lactate (D-PLA) and indole-3-lactate (ILA) [63,64]. The preparation of fermented cabbage involves mixing shredded cabbage with 2–3% (*w*/*w*) sodium chloride and incubating it at room temperature for 2–3 weeks with limited oxygen exposure. The combination of salt and low oxygen favors the growth of LAB. During fermentation, the dominant LAB species shift over time. Initially, heterofermentative species such as *Leuconostoc mesenteroides* and *Weissella* spp. predominate, producing lactic acid, acetic acid, carbon dioxide, and ethanol from glucose. As the environment becomes more acidic, conditions favor homofermentative LAB like *Lactiplantibacillus plantarum* and *Levilactobacillus brevis*, which produce lactic and acetic acids [65]. This ongoing acidification enhances the stability and preservation of sauerkraut [66,67].

Fermentation enhances the bioavailability of vitamins and minerals in cabbage, and generates antioxidants and probiotics, which contribute to the health advantages of sauerkraut [68,69].

Besides the antimicrobial effects and sensorial properties of fermented foods, lactic acid and acetic acids are associated with improvement of intestinal barrier function [70,71,72], immune function [73], and metabolic health (e.g., fasting blood glucose and insulin sensitivity) [72,74,75].

In addition, cabbage fermentation guided by LAB shows higher concentrations of phenolic compounds (polyphenols, phenolic acids) [76], carotenoids [77], glucosinolate breakdown products (ascorbigen, indole-3-carbinol, and isothiocyanates) [78,79], and other bioactive metabolites [80]. Intestinal barrier-protective properties following production of microbiota-associated D-PLA and ILA are the main advantages of the amino acid derivatives, D-PLA [81] and ILA [82,83,84,85]. The activation of signaling pathways involving PPAR-γ (D-PLA) and AHR (ILA) exerts these effects. PLA and ILA, along with lactic acid, are significant for the immunomodulatory, antioxidant, and anti-carcinogenic properties of fermented cabbage [79,80].

#### 2.6.2. Kefir

Kefir is a fermented drink with low alcohol content, acidic and bubbly from the fermentation carbonation of kefir grains with milk or water [86,87]. The starter is the kefir grains. Kefir grains range in size from 1 to 4 cm in length and look like small cauliflower florets in shape and color. This gelatinous and slimy structure is comprised of kefiran, a natural matrix of EPSs and proteins. In this matrix, LAB, yeast, and acetic acid bacteria (AAB) co-exist in a symbiotic connection [86].

The most abundant bacterial species in kefir grains are *Lactobacillus kefiranofaciens*, *Lacticaseibacillus paracasei*, *Lactiplantibacillus plantarum*, *Lactobacillus acidophilus*, and *Lactobacillus delbrueckii* subsp. *bulgaricus*. The predominant yeast species present in kefir are *Saccharomyces cerevisiae*, *S. unisporus*, *Candida kefyr*, and *Kluyveromyces marxianus* ssp. *marxianus* [88].

Kefiran is a postbiotic from kefir that has shown potential benefits in alleviating food allergies by modulating both the intestinal microbiome and the immune system [89]. This water-soluble branched glucogalactan postbiotic also showed antimicrobial and healing activity [90]. Other postbiotics from kefir LAB include surface layer proteins (SLPs) and various EPSs that may have a beneficial role in gut dysbiosis and obesity management [91].

Some of the most significant health benefits of kefir beverage consumption include anti-microbial, anti-tumor, anti-carcinogenic, and hypocholesterolemic effects. In addition, anti-hypertensive, anti-diabetic, and immunomodulatory activities, along with improved lactose digestion, are reported [92,93].

#### 2.6.3. Kimchi

Kimchi, a typical fermented Korean vegetable food, is considered one of the most important sources of postbiotics since it contains *Lactiplantibacillus plantarum*, a homofermentative LAB type producing organic acids, and *Leuconostoc mesenteroides*, a heterofermentative type producing carbon dioxide, acids, and weak alcohols [94]. The flavor (taste and aroma) of the product is controlled by these bacteria. Sugars are transformed into lactic acid, and this process characterizes yogurt, kimchi, and fermented cereals. This fermentation is primarily driven by LAB [95].

## 3. Mechanisms of Action of Postbiotics for Prevention and Management of Colorectal Cancer

It is well known that dietary intervention can play a great role in modulating human health and disease. Many bioactive compounds reported in the literature have been described to have significant benefits on CRC and modulate the gut microbiota [20]. As an example, Ou J et al. explored the impact of diet on colon cancer risk. They analyzed gut microbiota metabolites in individuals at high risk (African Americans) and low risk (rural native Africans) for colon cancer. Their research revealed significant links between a decreased production of SCFAs, elevated levels of secondary bile acid metabolites, and an increased risk of colon cancer [96]. Postbiotics may exert anti-carcinogenic and chemopreventive effects in CRC through a range of interrelated mechanisms involving modulation of epithelial cell signaling, immune and inflammatory responses, and microbial–host interactions. However, further larger studies are needed to fully elucidate their mechanisms and optimize their therapeutic potential.

### 3.1. Anti-Inflammatory and Immunomodulatory Effects

Chronic inflammation is a hallmark of cancer [97]. Several postbiotic compounds, particularly SCFAs, downregulate pro-inflammatory signaling cascades, acting as chemopreventive agents. Butyrate suppresses nuclear factor kappa B (NF-κB) activation, reducing the expression of pro-inflammatory cytokines like TNF-α, IL-6, and IL-1β. Similarly, other SCFAs may modulate the mitogen-activated protein kinase (MAPK) pathway [3,98]. Butyrate suppresses LPS-induced NF-κB activation via GPR109A in IEC models, in tissue samples of patients with CRC, and in mouse colon [99]. One study explored the role of EPSs in modulating inflammation. EPSs bind to the TLR2 receptor of intestinal epithelial cells, leading to the inhibition of IL-17 production and promotion of IL-10 expression [100].

Moreover, butyrate and SCFAs maintain intestinal immune homeostasis through the modulation of regulatory T cells (Tregs). The proliferation of CD4^+^ T lymphocytes is limited by Tregs, and they are therefore essential for suppressing inflammatory responses [101]. Butyrate also has a protective effect on the barrier [102,103].

The aryl hydrocarbon receptor (AhR), activated by indole derivatives, promotes the secretion of IL-22 and IL-10 while reducing the expression of pro-inflammatory cytokines such as TNF-α and IL-6 [104,105]. A study highlighted a novel mechanism in which *Lactobacillus reuteri* and dietary tryptophan interact to regulate gut immunity via AhR activation. *L. reuteri* produces indole derivatives from tryptophan, which activate AhR in CD4^+^ T cells. AhR activation leads to the downregulation of ThPOK, a transcription factor that enables CD4^+^ T cells to transition into DP IELs [106].

Anti-inflammatory and immunomodulatory functions are exerted by SLPs derived from various *Lactobacillus* species. SLPs isolated from *Propionibacterium fischeri* reduced TNF-α and IL-8 and inversely induced a significant increase in the expression level of TFG-β in HT-29 cells [107]. In a similar study, SLPs from *Lactobacillus helveticus* MMLh5 exerted anti-inflammatory effects by reducing the levels of both basal and induced NF-κ B in the presence of proinflammatory stimulus IL-1β in Caco-2 cell lines [108]. Moreover, SLPs from *Lactobacillus plantarum* reversed intestinal epithelial cell damage induced by pathogenic *E. coli* [109].

TAs play a role in immune modulation, leading to the simultaneous decrease in IL-12 and production of IL-10 in animal models. The effect of these TAs on IL-10 production was mediated by TLR2-dependent ERK activation [110]. Additionally, regulation of Treg T cell function is carried out along with suppression of intestinal inflammation, maintenance of homeostasis in the intestine, and exertion of antitumor and antioxidant effects [111]. Conversely, another study reported that LTA induces intestinal immune activation, resulting in the production of TNF-α and IL-12, which contribute to intestinal mucosal damage [112].

In an interesting study, it was shown that ILA, a metabolite of *Lactobacillus plantarum* L168, improved intestinal inflammation and dysbiosis and slowed down tumor growth. Experiments were carried out using adenocarcinoma cell lines and animal models. The production of IL12a in dendritic cells was promoted by ILA by facilitating the increase in H3K27ac binding to IL12a enhancer regions, thereby aiding in the activation of CD8^+^ T cell (cytotoxic T lymphocyte, CTL) responses against tumor development [113].

Yan et al. isolated and purified proteins secreted by the probiotic *Lactobacillus rhamnosus*. Two key proteins, p75 (75 kDa) and p40 (40 kDa), were used to treat human (HT-29) and mouse (YAMC) intestinal cells and murine ex vivo colon samples. The p75 and p40 proteins initiate the PI3K/Akt pathway, inhibit TNF-α-induced apoptosis and pro-inflammatory cytokines, stimulate the proliferation of intestinal epithelial cells, and reduce epithelial damage induced by TNF-α [114]. Bäuerl et al. demonstrated that the extracellular vesicles isolated from *L. casei* have the proteins p40 and p75 on their surfaces. T84 cells were treated with p40 and p75 vesicles, which induced EGFR phosphorylation in a dose-dependent manner, showing anti-inflammatory properties [115]. Five LAB strains from Korean kimchi were reported to have a protective role in inflammatory responses. The expression of VDR and autophagy was measured by HCT-116 and intestinal organoids with conditional medium (CM) from these strains. The LAB-CM-treated groups showed higher mRNA expression of *VDR* and its target gene cathelicidin compared with the control group [116].

### 3.2. Apoptosis Induction and Tumor Suppression

Postbiotics represent a promising frontier in cancer research due to their ability to induce apoptosis and suppress tumor growth through multiple biological pathways [117]. The mechanisms underlying their anticancer effects include activation of pro-apoptotic pathways, such as Bax and Bcl-2, regulation of p53, activation of Caspase 3 and 9, and suppression of tumor migration and invasion. As an example, the cytotoxic effects on HT-29 cells of two heat-killed probiotic bacteria were evaluated. *Lactobacillus Brevis* and *Lactobacillus paracasei* were isolated from a novel food from the Iranian diet ‘terxine’. Both bacteria inhibited proliferation and induced apoptosis of HT-29 cells, increasing the expression of mRNA of *CASP-3* and *CASP-9*, and reducing *BCL-2* [118]. Konishi et al. demonstrated in animal models that ferrichrome suppresses tumor growth both in vivo and in vitro by triggering apoptosis (activation of caspases) and inducing mitochondrial dysfunction [119].

The effects and mechanisms of butyrate-induced apoptosis in CRC models have been extensively investigated in several studies. Butyrate was shown to activate the intrinsic apoptotic pathway by upregulating pro-apoptotic proteins like Bax and downregulating anti-apoptotic proteins like Bcl-2 [120], and via the deactivation of mTOR/S6K1 signaling [121]. Similarly, Ryu et al. demonstrated that another SCFA, propionate, downregulated a histone arginine methyltransferase, PRMT1, in the HCT-116 cell line. This downregulation induced apoptosis by inhibiting phospho-p70 S6 kinase. To further validate the anticancer effect of propionate, cell growth assays using crystal violet staining were conducted (0 and 5 mM) in both normal enterocytes and the HCT-116 cell line. Notably, no significant effect of propionate treatment was observed in normal enterocytes [122].

### 3.3. Other Effects

Other chemopreventive and therapeutic effects of several postbiotics are described in the literature. Butyrate is a well-characterized HDAC inhibitor (epigenetic modulation) [123]. In CRC cells, it induces histone hyperacetylation, leading to the transcriptional activation of tumor suppressor genes, thereby promoting cell cycle arrest and apoptosis. The context-specific “butyrate paradox” illustrates this: while butyrate supports proliferation of healthy colonocytes, it inhibits proliferation and induces apoptosis of CRC cells due to altered metabolic states (Warburg effect) [124,125].

**Figure 3:**
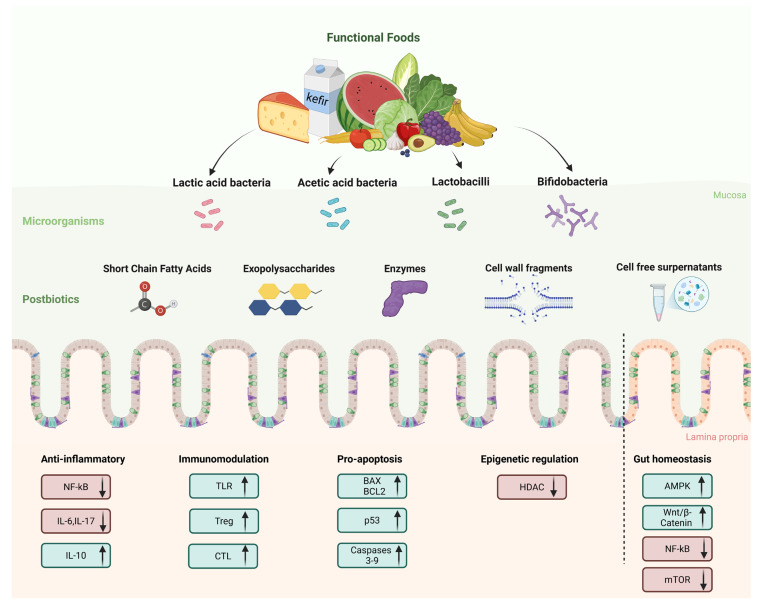
Postbiotics from functional foods: key mechanisms of action for prevention and management of colorectal cancer.

Other postbiotics from several *Lactobacillus* strains were shown to have a modulation role of gut barrier function, lipid metabolism regulation, and antioxidant activity [13,14,126,127]. A summary of the mechanisms of action of postbiotics in CRC is shown in Figure 3.

## 4. Therapeutic Potential and Application in Biomedical Systems: Current Evidence from Preclinical Studies

### 4.1. Methodologies and Software

In this section, the selection of the studies to be analyzed was carried out using the methodologies of an extensive literature search (ELS) and the principles of the PRISMA (The Preferred Reporting Items for Systematic reviews and Meta-Analyses) guidelines, 2020 version [128,129,130]. The initial searches were performed in three databases: PubMed, Scopus, and Web of Science. All keywords and terms were selected using the MeSH (Medical Subject Headings) vocabulary. The primary strings included “postbiotic AND colorectal cancer”, “postbiotic AND colorectal cancer OR tumor”, followed by secondary keywords in order to retrieve all studies, such as “butyrate”, “p40”, “p70”, “cell free supernatant”, “*Lactobacillus*”, “bacteriocins”, “exopolysaccharide”, “colon organoids”.

The literature review was conducted following the methodology outlined in the referenced guidelines. Following the removal of duplicate records, screening of titles and abstracts was carried out based on the inclusion and exclusion criteria detailed in Table 1. The search results from the ELS were imported into EndNoteWeb (Available online: https://web.endnote.com/, accessed on 15 March 2025), where initial selection was carried out using title and abstract screening, followed by full-text assessment. Only studies that satisfied the eligibility criteria proceeded to the next phase of the review, while those unrelated to the research focus or not meeting the inclusion standards were excluded.

**Figure 4:**
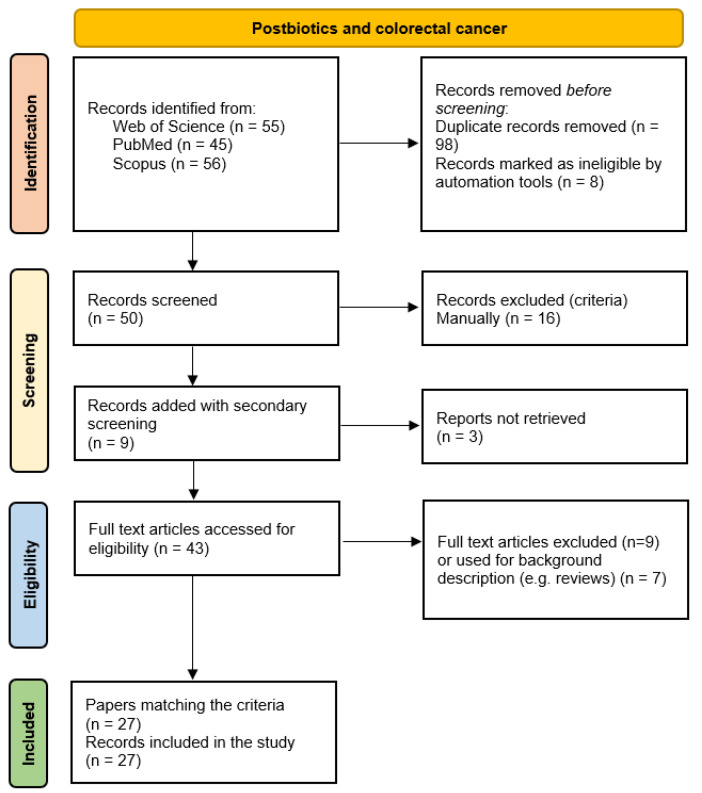
Flow diagram of the extensive literature search for studies on postbiotics and colorectal cancer.

The results obtained are summarized in Figure 4. The records were classified on the basis of the models used to conduct the study (in vitro, in silico, in vivo, ex vivo, advanced preclinical patient-derived models), postbiotics, and microorganisms of derivation. Key findings, pathways, and genes studied are reported. No clinical studies were found.

### 4.2. Studies on Cell Lines

A wide range of in vitro studies investigated the mechanisms of action and future possible benefits of postbiotics for CRC patients, including the production of organic acids, enzymes, peptides, and polysaccharides during the fermentation process. These studies provide mechanistic insights into how postbiotics may exert cytotoxic, pro-apoptotic, anti-proliferative, and immunomodulatory effects on CRC cell lines.

CFSs from *Lactobacillus casei* and *Lactobacillus rhamnosus* GG strains were reported to induce apoptosis and inhibit proliferation in HCT-116 cells by decreasing matrix metalloproteinase-9 (MMP-9) and increasing the tight junction protein zona occludens-1 (ZO-1) levels [131]. The CFSs were subsequently fractioned, and the most active fractions were identified (>100 kDa and 50–100 kDa). Similar results were obtained by Elham et al. for Caco-2 cells [132].

Chen et al. demonstrated that damage to HT-29 cell membranes was caused by the supernatants of seven strains of *Lactobacillus* at high concentrations. The PM177 strain showed the most potent inhibitory effect, followed by PM153 and BCRC14625. Among the strains studied, BCRC17010 stood out as having the best antitumoral potential, thanks to the induction of apoptosis, releasing lactate dehydrogenase (LDH), and producing nitric oxide (NO) [133].

Similarly, Jastrząb et al. identified *Lactococcus lactis* subsp. *lactis* Lc4 as a promising therapeutic strain capable of releasing arginine deiminase into its supernatant, which exerts strong anti-proliferative effects on CRC cells by depleting arginine and inducing cell cycle arrest [134].

Luo et al. investigated the mechanism of sodium butyrate (NaB)-induced autophagy in the CRC cell lines HCT-116 and HT-29, and their findings suggested that NaB treatment increased the formation of autolysosome and expression of phosphorylated liver kinase B1 (LKB1), AMP-activated protein kinase (AMPK), and acetyl-CoA carboxylase (ACC). In particular, LKB1 and AMPK are critical for NaB-mediated autophagy [135].

Recent studies have reported bacteriocins as postbiotic metabolites from *Lactobacillus plantarum* strains showing potent selective cytotoxicity effects via anti-proliferative mechanisms and induction of apoptosis in HT-29 cells without affecting healthy cells [136]. Selective inhibition of store-operated Ca^2+^ entry (SOCE) in CRC cells by downregulation of Orai1 and STIM1, thereby impairing cell migration, might also be caused by postbiotics from *Lacticaseibacillus paracasei* and *Lactiplantibacillus plantarum* [137].

Similarly, EPSs derived from *Lactobacillus acidophilus* were found to inhibit the growth of Caco-2 colon cancer cell line in a dose-dependent manner, both under normoxia and hypoxia [138].

Cousin et al. showed that induction of intrinsic apoptosis of HT-29 and HCT-116 cells, alone or enhanced by the activity of the apoptosis inducer, TNF-Related Apoptosis-Inducing Ligand (TRAIL), may be caused by propionate and acetate from *Propionibacterium freudenreichii* ITG-P9 [139].

In another study, LS174T human Dukes type B CRC adenocarcinoma cells were treated with butyrate, which is associated with beneficial probiotics (*Lactobacillus* and *Bifidobacterium*) species, at various concentrations. It positively influences mucin secretion with increased protein content (peak effects at 6 or 9 mM), which enhances the adherence of probiotic strains and stimulates the MAPK signaling pathway in intestinal cells, increasing gut defense [140]. CFSs from *Bifidobacterium longum* were also tested for anti-cancer properties in a study using Fn-secreted extracellular vesicles (Fnev)-infected CRC cells, with controversial results [141].

A preliminary in silico analysis guided the in vitro studies of Erfanian et al., which reported that *Lactobacillus acidophilus* postbiotics may exert their anti-proliferation and anti-migration activities via the Wnt signaling pathway (RSPO2, NGF, MMP7, SFRP1, SFRP2, SFRP4, and MMP7) [142]. The same authors obtained similar results in another study on postbiotics from *Bifidobacterium breve* and *Lactobacillus rhamnosus* [143].

Another study evaluated the safety and antimicrobial and anticancer effects of cell-free metabolites from *Gluconobacter oxydans* strains, isolated from Kombucha, as potential postbiotics. Using five AAB strains and three human cell lines, including HT-29, the results confirmed the safety and functional potential of selected AAB strains. The KNS30 strain showed the strongest activity against gastric cancer [144].

While these in vitro studies provide important mechanistic data, the models used (standard immortalized CRC cell lines) present notable limitations. Caco-2 cells, for instance, exhibit low expression of key phase I and phase II metabolic enzymes, including cytochrome P450s and glutathione S-transferases, which compromise their ability to replicate xenobiotic metabolism and bioactivation processes observed in vivo. Therefore, findings from these models should be interpreted with caution and ideally integrated and validated using more physiologically relevant systems, such as human-based co-cultures, 3D spheroids, organoid models, or animal studies, including xenograft models.

### 4.3. In Vivo Studies

The effectiveness of postbiotics in CRC prevention and therapy in vivo has been evaluated using several animal studies. Although limited, in vivo studies provide complementary evidence of postbiotic efficacy in CRC models. As an example, early-life supplementation with p40, a protein derived from *Lactobacillus rhamnosus GG*, was shown to enhance intestinal development and immune function in mice. Delivered via hydrogels, p40 promotes epithelial growth, tight junction formation, and IgA production through EGFR activation. These effects are absent without EGFR, highlighting its key role. Early p40 treatment also improves resistance to gut injury and inflammation in adulthood, suggesting long-term health benefits from probiotic-derived factors [145].

Sharma and Shukla reported the mitigation of early-stage colon cancer development in Sprague–Dawley rats by the CFS of *Lactobacillus rhamnosus* MD 14 MH656799, which includes acetamide, acetate, propionate, butyrate, thiocyanic acid, and oxalic acid compounds. The protective effects were associated with a reduction in fecal procarcinogenic enzyme activity, oxidative stress, and aberrant crypt foci, alongside the suppression of oncogenes like *β-catenin*, *K-ras*, *Cox-2*, and *NF-κB*, and the upregulation of the tumor suppressor gene *TP53*, resulting in near-normal colon tissue structure [146].

*Lactobacillus casei* ATCC334 supernatant, where ferrichrome was subsequently identified as the responsible molecule inducing apoptosis in CRC cells, exhibited minimal effect on normal intestinal epithelial cells while having stronger antitumor activity than conventional CRC drugs (5-FU and cisplatin). The findings were confirmed using xenograft models obtained by injecting SW620 cells into male BALB/c nude mice [119].

EPS application in postbiotics therapy was investigated by Ma et al., suggesting that oral administration of *Lactiplantibacillus plantarum*-12 EPSs to mice can reduce colon cancer symptoms through gut microbiota and metabolite modulation [147].

Furthermore, a new type of therapeutic strategy could lead to the use of bacterial lysates, given that *L. acidophilus* cell lysates, combined with immunotherapeutic antibodies, anti-CTLA-4, may help in forming the anticancer immune response in CRC-induced murine models. The combined administration leads to meaningful inhibition of increased amounts of proteobacteria and somewhat modulates the CRC-induced dysbiosis [148].

In a promising and advanced preclinical study, Lee et al. characterized the synergic effect of MS-20 Symbiota^®^, a mixture of microbial metabolites generated from fermentation of a soybean-based medium with multiple strains of probiotics and yeast, with anti-PD1 antibody therapy in xenograft mouse models, showing enhanced efficacy against tumor growth. This study also used fecal samples from CRC patients for ex vivo treatment to confirm the modulation of immune checkpoint inhibitor (ICI)-responsive bacteria [149]. A similar study using an AOM/DSS mouse model of CRC investigated the role of the potential postbiotic putrescine, finding that it reduced the number and size of colonic tumors and downmodulated the release of inflammatory cytokines in the colonic lumen [150].

Other studies tested the anti-proliferative/migration effect modulated by apoptosis, cell cycle arrest, and autophagy. For example, Zhong et al. evaluated the postbiotic MZY531 and its tumor growth inhibition effect in a xenograft mouse model through Bax/Bcl2/caspase-3 and JAK2/STAT3-mediated apoptosis and PI3K/AKT/mTOR and TGF-β/SMAD4-mediated autophagy and found interesting results [151].

### 4.4. Investigating Postbiotic Safety and Effects Using Advanced Preclinical Models

Conventional in vitro 2D cell cultures and co-culture systems were extensively used in the last few decades to predict cell behavior, morphology, physiology, and molecular responses. However, these systems lack the tissue complexity, cell-to-cell and cell-to-ECM interactions, and the physical stimuli required in a model for studying the unique events involved in cancer development and progression and associated treatments [152]. For this reason, studies based on animal models continue to be the gold standard, despite their notable drawbacks, including interspecies differences and ethical concerns. To address the gap between 2D in vitro systems and in vivo animal studies, advanced technologies such as 3D organoids and organ-on-chip (OoC) platforms have been developed, offering more physiologically relevant alternatives. They show great potential and broad applicability in drug development, safety assessment, personalized medicine, and advanced preclinical/clinical research. Organoids, self-organizing 3D structures derived from pluripotent stem cells or adult progenitor cells, recapitulate many key functions of the original tissue, including cellular heterogeneity and spatial organization [153,154]. Organoids derived from cancerous tissue (tumoroids) are particularly valuable for modeling tumor biology and drug responses [155,156,157,158]. However, cellular self-organization alone is insufficient to fully mimic native tissue architecture, which is why biomaterials such as hydrogels or decellularized scaffolds are employed to simulate ECM characteristics and support cell–cell communication [159,160,161]. The OoC system has been made possible thanks to the development of the lithography technique and, subsequently, the 3D printing technique. It consists of a silicon-based organic polymer, polydimethylsiloxane (PDMS), a microfluidic device designed to maintain cell culture in a closed environment and analyze cell biological characteristics. These technologies introduce the possibility of regulating fluidic parameters such as flow, pressure, oxygen, and pH in real time during the entire experimental procedure [162]. Colon organoids and tumoroids have been successfully developed and expanded, and long-term culture has been described in many studies [163,164]. Given the importance of gut microbiota, some models involve microinjecting bacteria into the organoid lumen to replicate host–microbe interactions more accurately [165]. This approach has also been used to study infections in other tissues, such as *Cryptosporidium* in the small intestines [166] and endometrial infections [167]. Combining organoids with microfluidic chips (organoid-on-chip systems) add greater experimental control, allowing real-time regulation of flow, oxygen, and mechanical forces. These platforms better simulate the intestinal environment, including peristalsis and crypt architecture [168].

In this context, the studies on postbiotics carried out using these advanced models are characterized by higher human relevance. Table 2 reports relevant results from the systematic review (SR) and ELS research, focusing on strings that include primary and secondary research keywords.

Although the majority of preclinical studies still rely on 2D cultures and mouse models, some combine organoids, animals, and microfluidics to investigate inflammation, epithelial regeneration, and tumor growth. Only a few studies utilize microfluidic chip systems. The main application was the production of hydrogel-based microspheres for postbiotic delivery. Several studies combined early use of cell lines, followed by integration of in vivo and organoid models. For example, Sugimura et al. found that *Lactobacillus gallinarum*, through the production of bioactive metabolites like ILA, significantly reduced tumor number and size compared to controls in mouse models. Its culture supernatant suppressed CRC cell proliferation and induced apoptosis in CRC cells and patient-derived organoids (but not normal cells). It also altered gut microbiota to create a more beneficial composition. The study was limited to two patients for organoid derivation [169]. However, the lack of standardized, interconnected systems limits data integration and translation across platforms. Harmonized models could reduce experimental time and cost, while offering closer alignment with human biology. These studies investigated the inflammatory modulation properties of several postbiotics. Cho Y. et al. focused on the *COX-2* gene pathway, while Lee H. et al. focused on (IFNγ)/TNFα, IL-1β, IL-6, IL-8, IL-10, and TGF-β pathways [170,171].

Few studies have been conducted on the maintenance/recovery of intestinal epithelium integrity and functionality following inflammatory stress. Furone et al. analyzed *Lactobacillus rhamnosus* postbiotic and its protective role on the alteration induced by gliadin in celiac disease patient-derived organoids. They found that the postbiotic acted on mTOR, inflammation, and autophagy pathways, all essential for the maintenance of the epithelium’s integrity and functionality. Although the model was not developed for CRC, the mTOR pathway, in particular the PI3K/Akt/mTOR pathway, was extensively described as a potential target in this type of cancer [172].

Finally, an advanced preclinical model based on human iPSC-derived intestinal epithelial cells (IECs) was used to assess the anti-inflammatory properties of heat-killed *Lactiplantibacillus plantarum* WCFS1 against the conventional Caco-2 cell model, offering improved physiological relevance [173].

## 5. Clinical Evidence, Formulation, and Delivery of Postbiotics

### 5.1. Clinical Evidence

While some large clinical trials were described for probiotics in post-surgical CRC, no clinical trials have been reported for postbiotics application in CRC patients. For example, Zaharuddin et al. showed that probiotics containing *Lactobacillus* and *Bifidobacteria* strains (*Lactobacillus acidophilus* BCMC^®^ 12130, *Lactobacillus lactis* BCMC^®^ 12451, *Lactobacillus casei* subsp. BCMC^®^ 12313, *Bifidobacterium longum* BCMC^®^ 02120, *Bifidobacterium bifidum* BCMC^®^ 02290, *Bifidobacterium infantis* BCMC^®^ 02129) are safe to be consumed by CRC patients four weeks after surgery and reduced pro-inflammatory cytokines, probably by intestinal microenvironment modification [174]. In another randomized controlled prospective trial, a probiotic formulation containing eight bacterial cultures (*Lactobacillus acidophilus*, *Lactobacillus casei*, *Lactobacillus plantarum*, *Lactobacillus rhamnosus*, *Bifidobacterium lactis*, *Bifidobacterium bifidum*, *Bifidobacterium breve*, *Streptococcus thermophilus*) was used to treat CRC patients, and its use led to a statistically significant reduction in postoperative complications [175]. These authors reported that patients getting probiotics spent fewer days hospitalized and had a lower risk of infection. There was a notable reduction in complications for tumors located in the ascending colon and rectum. Additionally, during the first six months after surgery, the “probiotic group” experienced fewer rare fatal consequences [175].

In another interesting clinical study, different levels of SCFA and HSP 70 expression in CRC patients were compared with those in non-CRC patients. Fauzi et al. found that CRC patients had lower levels of acetate, propionate, and butyrate acids compared with non-CRC patients and that short-chain fatty acids were indirectly correlated with CRC pathogenesis [176]. The investigation revealed that, compared to non-CRC individuals, CRC patients had lower SCFAs levels. The results propose a probable relationship between decreased SCFA concentrations and CRC progression. In the placebo-controlled RIBOGUT trial, Liu et al. found that oral supplementation with 100 mg/day riboflavin for 2 weeks increased the number of *F. prausnitzii* in feces, promoting butyrate production in the absence of major shifts in gut microbiota composition, while the complexity and stability of the bacterial network were enhanced [177].

Another study evaluated the potential benefits of administering probiotics postoperatively to reduce gastrointestinal complications and gut microbiota disturbances in CRC patients undergoing chemotherapy. The use of *Bifidobacterium infantis*, *Lactobacillus acidophilus*, *Enterococcus faecalis*, and *Bacillus cereus* helped protect against chemotherapy-induced dysbiosis and promoted the production of SCFAs [178].

Although no clinical studies specifically investigating the administration of postbiotics in CRC patients were identified, existing evidence suggests that supplementation with these metabolites may have beneficial effects. However, large-scale clinical trials are urgently needed to address this gap and validate their therapeutic potential.

### 5.2. Postbiotic Formulation and Delivery

Although the preventive, palliative, and therapeutic roles of several postbiotics were extensively discussed, their clinical potential may be limited by instability, rapid degradation, and poor bioavailability. To overcome these challenges, innovative drug delivery systems have led to the targeted delivery of postbiotics to their sites of action, resulting in improved therapeutic efficacy and reduced side effects. This type of pharmaceutical approach is also useful for masking unpleasant organoleptic properties and increasing patients’ compliance [179].

Several novel pharmaceutical formulations for enhancing the delivery of butyrate and other SCFAs to tumor cells were studied and described in the literature [180]. In this line, refined formulation and delivery of postbiotics may enable targeted therapeutic modulation of these underlying mechanisms. Advanced drug delivery strategies, such as enteric coating, conjugation with dietary fibers, prodrug design, and nanoformulations, enhance the stability of metabolites during bodily transit, ensuring targeted delivery to specific sites. These systems also improve release profiles, helping to prolong the duration of therapeutic effectiveness. As an example, NaB was found to inhibit the growth of a variety of other cancer cell lines in vitro. For this purpose, a combination of poly (lactic-co-glycolic acid) (PLGA) and poly (N-isopropylacrylamide) (PNIPAM) was used to create NaB-loaded microspheres, which showed slow degradation, prolonged retention, and controlled release, making them effective for treating different clinical conditions [181]. The established formulation can enhance cardiac function in acute myocardial infarction by triggering the mitochondrial protein Sirt3. The organized release system can improve the bioavailability and therapeutic effect of NaB, emphasizing the significance of advanced delivery approaches in postbiotic-based treatments.

Enteric encapsulations, prodrugs, and esterification with dietary fibers have been employed to ensure postbiotics reach the distal gut, where they exert their beneficial effects. As microbial metabolites are normally produced in the distal gut, targeting their delivery to this site enables them to mimic their production by an eubiotic microbiome. Enteric coatings protect drugs from gastric degradation and can also impart delayed-, modified-, and/or controlled-release characteristics by virtue of their selective disintegration at specific pH values or upon exposure to intestinal enzymes [182]. Usually, prodrugs are designed to delay metabolite release until they reach systemic circulation, improving stability, bioavailability, and duration of action of small-molecule drugs [183]. Postbiotic-prodrug formulations are currently only associated with SCFA prodrugs, which, in turn, predominantly comprise butyrate prodrugs. In this context, formulations like Tributyrin (TB), Pivaloyloxymethyl butyrate (AN-9, Pivanex), Butyroyloxymethyl diethylphosphate (AN-7), and N-(1-carbamoyl-2-phenyl-ethyl) butyramide (FBA) have improved the pharmacokinetic characteristics [123,184,185,186]. Additionally, esterification with dietary fibers represents another pharmaceutical approach for colon-targeted delivery, prolonging the release of short-chain fatty acids (SCFAs), eliminating their unpleasant organoleptic properties, and supporting gut microbiota interactions [187]. For example, SCFA-esterified HAMS (High amylose maize starch) selectively increases SCFA levels in the distal GI tract and alters gut microbiota, expanding SCFA-utilizing bacteria and shifting microbial metabolism toward SCFA utilization [188,189,190]. Offering a promising approach to extend drug exposure profiles, nanoformulations act as targeted-delivery carriers, concentrating drugs at specific sites, or as controlled-release systems, maintaining therapeutic drug levels over an extended period. Nano-scale carriers reduce the need for frequent dosing and improve stability against gastric degradation, offering another strategy for controlled postbiotics release in the lower intestine. Cholesteryl butyrate-loaded solid lipid nanoparticles (Chol-But SLNs) provide stability in acidic gastric conditions, enable sustained drug release, and support targeted delivery to inflamed tissues through preferential uptake by immune cells [191]. Polyvinyl butyrate nanoparticles (PV-But NPs) were designed for controlled butyrate delivery to the lower intestine, resisting pancreatic lipase hydrolysis and enabling slow butyrate release [192]. Liposome-encapsulated sodium butyrate (NaB-Lip), originally developed to address *F. nucleatum*-induced chemotherapy resistance, accumulates in *F. nucleatum*-infected colorectal tumors and, when combined with oxaliplatin, significantly reduces tumor growth and improves survival [193].

Certain postbiotics may help alleviate colonic inflammation and support the restoration of gut barrier function. For instance, p40, a protein produced by *Lactobacillus rhamnosus* GG, has been shown to counteract cytokine-induced epithelial cell apoptosis and prevent disruption of the intestinal barrier. Targeted delivery of hydrogel-coating p40 (to protect p40 from degradation) is effective in preventing and treating intestinal injury and inflammation, as well as promoting protective immune response [194,195]. HM0539, a secreted protein identified by means of liquid chromatography–tandem mass spectrometry analysis from the culture of *L. rhamnosus GG*, plays a protective role in maintaining the integrity of the intestinal barrier by increasing the expression of intestinal mucin and preventing intestinal barrier injury. A pectin/zein beads delivery system was used to deliver HM0539 to the colon, as they protect HM0539 from protease attack. In this study, its potential bioactivity was evaluated in vitro and in vivo [196].

Moreover, oral codelivery of probiotic *Lactobacillus acidophilus* biofilm and postbiotic indole-3-propionic acid has been reported by Fang et al. to alleviate colitis in mice and reduce chronic inflammation. The authors used a microfluidic-based montmorillonite composite microparticle system, which was selected to support biofilm formation. This system enhances stability against gastrointestinal stress, improves mucosal adhesion, and provides controlled, colon-targeted release. In a colitis mouse model, the codelivery system effectively reduced inflammation, repaired the gut barrier, and modulated microbiota. It highlights how postbiotics, when strategically delivered, can synergize with probiotics, suggesting translational relevance for CRC therapy [197].

In addition, targeted delivery systems using prebiotic carbohydrate matrices have been reported by Zhang et al. [198,199], focusing on structures, release mechanisms, and applications. Encapsulation of bioactive ingredients into nanohydrogels, nanoparticles, nanoemulsions, micro/nanocapsules, and nanofibers to achieve controlled/targeted delivery is the key step. Of course, postbiotics are produced by prebiotic-based delivery systems, which degrade in the posterior colon, thereby leading to the exertion of prebiotic functions and beneficial effects on host health.

Finally, Cuevas-González et al. [200] and Monteiro et al. [201], along with the recent review by Taskoparan et al. [202], also refer to the health-promoting mechanisms of postbiotics and paraprobiotics.

## 6. Conclusions and Future Perspectives

The increasing amount of research discussed in this work evidences the promising role of postbiotics as co-adjuvant agents in the therapy and management of CRC. Postbiotics from functional foods, including short-chain fatty acids, exopolysaccharides, enzymes, cell wall fragments, and other microbial metabolites, exhibit a broad spectrum of health-promoting activities, such as anti-inflammatory, immunomodulatory, antioxidant, and antitumor effects. These mechanisms collectively contribute to intestinal barrier protection, immune homeostasis, and inhibition of CRC progression through apoptosis induction, modulation of oncogenic signaling pathways, and epigenetic regulation.

Through comprehensive mapping of the literature following PRISMA and ELS guidelines, this review uniquely categorizes research according to the experimental models employed and provides a more mechanistic, structured, and translationally relevant methodological framework. Preclinical studies, particularly those employing advanced models such as organoids and organ-on-chip systems, demonstrate the capacity of postbiotics to emulate host–microbe interactions with greater physiological relevance than traditional 2D cultures. These models yield critical insights into the multifaceted bioactivities of postbiotics; however, their widespread adoption remains limited due to a lack of standardization and integration into translational pipelines. Despite promising preclinical data, the clinical application of postbiotics in CRC remains in its early stages. Persistent challenges include the need for standardized definitions, scalable manufacturing protocols, comprehensive safety assessments, and robust clinical validation. Additionally, regulatory pathways for the approval and therapeutic integration of postbiotic-based interventions require clearer delineation.

Future research should aim to translate preclinical efficacy into clinical settings through well-designed and large-scale human trials. Precision medicine strategies will be essential to tailor postbiotic therapies to individual microbiome profiles and host genetics. Additionally, the integration of multi-model platforms, interdisciplinary collaboration, and advanced pan- and multi-omic technologies will be critical to establish robust, data-driven, and patient-centered therapeutic approaches. With continued interdisciplinary effort, postbiotics hold strong potential to become effective, safe, and sustainable adjuncts in CRC prevention and treatment strategies.

## Figures and Tables

**Table 1:** Inclusion and exclusion criteria for systematic review and extensive literature search.

Criterion	Decision	
	Inclusion	Exclusion
Default keywords and search terms exist as a whole or at least in the title, keywords, or abstract of the article	×	
The article is published in a peer-reviewed scientific journal	×	
The article is written in English	×	
Studies where terms such as prebiotics were referred to; however, supernatant/heat-killed cultures were used for testing/assessing	×	
Studies on diseases considered high risk factors for colorectal cancer, and a relevant study model was developed/used	×	
Duplicate records		×
The full text is not available		×
Articles published before 2010		×
Only testing live microorganisms		×
Studies on gut microbiota transplantation		×

**Table 2:** Studies on postbiotics and colorectal cancer.

ID	Model	Study Type	Microorganism Strain/Species	Molecules of Interest	Events	Pathway/Gene Involved	Notes	Ref
1	HCT-116	in vitro	*Lactobacillus casei* and *Lactobacillus rhamnosus GG*	Cell-free supernatant	Decreasing matrix metalloproteinase-9 (MMP-9) and increasing the tight junction protein zona occludens-1 (ZO-1) levels	cell invasion		[131]
2	HT-29	in vitro	7 strains of *Lactobacillus*	Cell-free supernatant	Lactate dehydrogenase regulation	apoptosis		[133]
3	HCT-116 HT-29	in vitro	*synthetic*	sodium butyrate	Autophagy	LKB1–AMPK pathway		[135]
4	HT-29	in vitro	*Lactobacillus* *plantarum*	bacteriocins	Antiproliferative effect	apoptosis	study using several cancer cell lines	[136]
5	Caco-2	in vitro	*Lactobacillus* *acidophilus*	exopolysaccharide	Upregulation of the expression of *PPAR-γ*			[138]
6	HT-29 HCT-116	in vitro	*Propionibacterium freudenreichii*	culture supernatant, metabolites (propionate/acetate)	Increased pro-apoptotic gene expression (*TRAIL-R2*/*DR5*) and decreased anti-apoptotic gene expression (*FLIP*, *XIAP*); death receptors (TRAIL-R1/DR4, TRAIL-R2/DR5) and caspases (caspase-8, -9, and -3) activation; Bcl-2 expression inhibition	extrinsic apoptotic pathway	in combination with TNF-Related Apoptosis-Inducing Ligand (TRAIL)	[139]
7	LS174T	in vitro	*Lactobacillus acidophilus* and *Bifidobacterium longum*	butyrate	dose-dependent increase in mucin protein contents; increased transcriptional levels of *MUC3*, *MUC4*, and *MUC12*	MAPK signaling pathway	doses: 6 or 9 mM	[140]
8	scRNA-seq analysis and DEGs analysis HT-29 human dermal fibroblasts	in silico in vitro	*Lactobacillus acidophilus ATCC4356*	cell-free supernatant	Cell cycle arrest at G1 phase, anti-proliferative and anti-migration effects, and anti-proliferative activity on control fibroblasts.	Wnt signaling (SFRP1, SFRP2, SFRP4, MMP7)		[142]
9	HT-29 human dermal fibroblast	in vitro	*Bifidobacterium breve Lactobacillus rhamnosus*	cell-free supernatant	Anti-proliferation, anti-migration, and apoptosis-related effects	apoptosis: Bax/Bcl2/caspase-3; Wnt signaling: RSPO2, NGF, MMP7		[143]
10	Caco-2	in vitro	*Lactobacillus casei*	cell-free supernatant	Tumor cell cytotoxic effect	apoptosis	comparison of probiotic (live), paraprobiotic (heat-killed), and postbiotics (CFS)	[132]
11	HT-29	in vitro	*Gluconobacter oxydans* strains isolated from Kombucha (KNS30, KNS31, KNS32, K1, and K2)	gluconic acid, glucuronic acid, acetic acid, pyruvic acid, fumaric acid, and lactic acid	Tumor cell cytotoxic effect	apoptotic/necrotic: annexin V and PI positive	study using gastric cell line: AGS; HUVEC cell lines used as control	[144]
12	HT-29 HCT-116	in vitro	*Lactobacillus lactis*	cell-free supernatant	Depletion of arginine, decreased levels of c-Myc, and reduced phosphorylation of p70-S6 kinase	cell cycle arrest		[134]
13	NCM460 Caco-2 HT-29	in vitro	*Lacticaseibacillus paracasei* and *Lactiplantibacillus plantarum*	heat-inactivated cultures	Downregulation of Orai1 and STIM1	FAK pathway (Store-operated calcium entry)		[137]
14	HT-29	in vitro	*Saccharomyces boulardii*	cell-free supernatant	Increased expression of *Caspase 3* and *PTEN* genes; decreased expression of *RelA* and *Bcl-XL* genes	apoptosis		[117]
15	HT-29 Fnevs infection model	in vitro	*Bifidobacterium longum*	cell-free supernatant	Inhibition of proliferation, migration, and invasion	inhibitory effects on the expression of specific oncogenes (e.g., *Myc*, *IL16*, *KCNN2*, *ACSBG1*, *Pum1*, *MET*, *NR5A2*)	controversial results	[141]
16	mouse colon carcinoma CT26.WT tumor cells were injected subcutaneously into BALB/c mice	in vivo	*Weizmannia coagulans* MZY531	powder of *W. coagulans* MZY531; oligosaccharide suspension	Inhibition of tumor growth by modulating apoptosis and autophagy in tumor cells	apoptosis: Bax/Bcl2/caspase-3 and JAK2/STAT3 autophagy: PI3K/AKT/mTOR and TGF-β/SMAD4		[151]
17	Sprague–Dawley rats	in vivo	*Lactobacillus rhamnosus* MD 14	metabiotic extract (acetate, butyrate, propionate, acetamide, thiocyanic acid, and oxalic acid)	Downregulation of oncogenes (*K-ras*, *β-catenin*, *Cox-2*, *NF-κB*) and upregulation of the *TP53* gene leading to almost normal colon histology	Wnt/β-Catenin Pathway	active components in the metabiotic extract were characterized by LC-MS	[146]
18	xenograft mouse model CT-26 cells subcutaneously injected into BALB/c mice	in vivo ex vivo	multiple strains of probiotics and yeast	MS-20 “Symbiota^®^” in combination with anti-programmed cell death 1 (PD1) antibody	Inhibited colon and lung cancer growth	CD8+ T cells and PD1 expression	fecal samples from six patients were used for ex vivo evaluation	[149]
19	C57BL/6 mouse model where cancer was induced via AOM/DSS administration	in vivo	*Escherichia coli* Nissle 1917	putrescine	Inhibition of the growth of the pathogenic strain pks+ *E. coli* NC101; reduced the number and size of colonic tumors, regulation of inflammatory cytokines; shift in the composition of gut microbiota	cell proliferation; fecal Lcn-2 marker of inflammation in inflammatory bowel diseases, TNFα, IL6, and IL10; 16S rRNA amplicon sequencing		[150]
20	xenograft models obtained by injecting SW620 cells into male BALB/c nude mice Caco-2/bbe SKCO-1 SW620	in vivo in vitro	*Lactobacillus casei* ATCC334	ferrichrome	Activation of the JNK-DDIT3-mediated apoptotic pathway	JNK-DDIT3-mediated apoptotic pathway	effect of ferrichrome was compared with 5-FU and cisplatin	[119]
21	C57BL/6 mouse model where cancer was induced via AOM/DSS administration	in vivo	*Lactiplantibacillus plantarum*-12	exopolysaccharide	Activation of caspase cascade and NF-κB signaling (IκB-α, p65, p-p65, p38, and p-p38)	inflammatory signaling and apoptosis	additional untargeted fecal metabolomic analysis	[147]
22	BALB/c mice CRC models induced via AOM/DSS administration	in vivo	*Lactobacillus acidophilus*	lysates	Increased CD8 + T cells and effector memory T cells, decreased Treg and M2 macrophages	TLR signaling pathway	combination with CTLA-4-blocking antibodies	[148]
23	C57B/6 mouse model CRC cell lines Organoids from CRC patients	in vitro in vivo organoids	*Lactobacillus gallinarum*	cell-free supernatant (indole-3-lactic acid, most enriched metabolite)	Antitumorigenic role: proliferation, apoptosis, cell cycle distribution, gut microbiota modulation	cell proliferation apoptosis		[169]
24	Organoids derived from C57BL/6 male mice small intestines and colon	in vitro in vivo organoids	*Lactiplantibacillus plantarum* KM2 and *Bacillus* *velezensis* KMU01	cell-free supernatant	Inflammatory response; LPS-induced and mitochondrial homeostasis through mitophagy and mitochondrial biogenesis	COX-2 decreased; expression of tight-junction markers ZO-1, claudin, and occludin increased, and expression of mitochondrial homeostasis factors PINK1, parkin, and PGC1a also increased.		[170]
25	hPSC-derived intestinal organoids C57BL Mice Caco-2	in vitro in vivo organoids	*Limosilactobacillus reuteri* DS0384	N-carbamyl glutamic acid (NCG)	Intestinal epithelial maturation, inflammatory response, and intestinal epithelial barrier integrity	mature specific marker: (CDX2), (OLFM4), (DEFA5 and LYZ), (KRT20, CREB3L3, DPP4, LCT, SLC5A1, and MUC13); Inflammatory pathway: (IFNγ)/TNFα, IL-1β, IL-6, IL-8, and TNFα; localization of zonula occludens-1		[171]
26	Caco-2 Organoids derived from biopsies of a celiac disease patient	in vitro organoids	*Lactobacillus rhamnosus GG*	cell-free supernatant	Alteration in autophagy and inflammation pathways induced by gliadin in celiac disease	mTOR pathway: phosphorylation of p70S6K, p4EBP-1; inflammatory marker: NF- kb; autophagy: LC3II and p62 protein, SQSTM1 autophagosome membrane marker		[172]
27	Caco-2 hiPSC-derived IEC monolayers	in vitro advanced patient-derived in vitro	*Lactiplantibacillus plantarum*	heat-killed	Inflammatory response	IL-8, REG3α, and HBD2		[173]

## Data Availability

No new data were created or analyzed in this study.
